# Pterostilbene 4′-*β*-Glucoside Protects against DSS-Induced Colitis via Induction of Tristetraprolin

**DOI:** 10.1155/2017/9427583

**Published:** 2017-05-07

**Authors:** Yingqing Chen, Jeongmin Park, Yeonsoo Joe, Hyeok-Jun Park, Seung-Joo Jekal, Daisuke Sato, Hiroki Hamada, Hun Taeg Chung

**Affiliations:** ^1^Department of Biological Sciences, University of Ulsan, Ulsan 680-749, Republic of Korea; ^2^Department of Clinical Laboratory Science, Wonkwang Health Science University, Iksan 570-750, Republic of Korea; ^3^Department of Life Science, Okayama University, Okayama 770-0005, Japan

## Abstract

Pterostilbene, a dimethyl ester analog of resveratrol, has anti-inflammatory and antioxidative effects and alters cell proliferation. Tristetraprolin (TTP) promotes the degradation of proinflammatory mediators via binding to adenosine and uridine- (AU-) rich elements (ARE) located in the 3′-untranslated regions of mRNAs. Here, we utilized pterostilbene 4′-*β*-glucoside (4-PG), a compound derived from pterostilbene, to investigate whether it has anti-inflammatory effects on dextran sulfate sodium- (DSS-) induced colitis via TTP enhancement. TTP expression was increased in 4-PG dose- and time-dependent manners in RAW264.7 cells. The production of proinflammatory cytokine, such as TNF-*α*, was reduced by 4-PG in vitro. To investigate the role of TTP in the anti-inflammatory effects of 4-PG, we used DSS–induced colitis in TTP WT and KO mice as models. The expression levels of TTP and proinflammatory cytokines were determined in serum and colon tissue. 4-PG increased the expression of TTP while suppressing proinflammatory cytokines both in vitro and in vivo. These findings suggest that treatment with 4-PG mediates the anti-inflammatory effects of 4-PG on DSS-induced colitis via enhancing TTP expression.

## 1. Introduction

Inflammatory bowel diseases (IBD), including ulcerative colitis and Crohn's disease, are chronic and recurrent intestinal inflammatory disorders caused by the transmural infiltration of neutrophils, macrophages, lymphocytes, and mast cells, ultimately giving rise to mucosal dysfunction and ulceration [1]. Although the etiology of IBD remains unclear, it is widely believed that the overproduction of proinflammatory cytokines and multiple genetic defects is associated with IBD [2]. Dietary polyphenols, including curcumin, quercetin, and resveratrol, show protective effects in IBD by reducing the inflammatory response and enhancing antioxidant defense [3]. In particular, resveratrol, a potent anti-inflammatory agent, was shown to increase the activities of superoxide dismutase (SOD) and glutathione peroxidase (Gx) and decrease the expression levels of TNF-*α*, IL-8, IFN-gamma, p22 (phox), and gp91 (phox). As a result, the severity of IBD was inhibited in the resveratrol-treated mice [4]. Pterostilbene (3,5-dimethoxy-4′-hydroxystilbene) is a phytoalexin and a natural dimethylated analog of resveratrol (3,5,4′-trihydroxystilbene) [5]. Pterostilbene has the anti-inflammatory action by inhibiting inducible nitric oxide synthase (*iNOS*) and cyclooxygenase-2 (*COX-2*) inflammatiory genes via p38 inactivation [6]. Also, pterostilbene possesses anticarcinogenic properties which may be responsible for the cancer chemopreventive potency of pterostilbene [7]. Additionally, pterostilbene can significantly decrease dextran sulfate sodium- (DSS-) induced murine aberrant colon crypt foci and tumor formation through anti-inflammatory and antioxidant mechanisms [8]. The stilbenes, such as resveratrol [9], pterostilbene [10], and piceatannol [11], have been explored as means to potentiate the antiaging, antioxidative, and antitumorigenic effects. Glycosylation of pterostilbene results in high-phosphodiesterase (PDE) inhibitory activity [12]. Also, the levels of PDE isoforms are upregulated in Alzheimer's disease (AD) patients [13]. Therefore, 4′-*β*-glucoside of pterostilbene as PDE inhibitors could be a potential chemopreventive agent for AD therapy [14].

The underlying mechanism(s) of pterostilbene 4′-*β*-glucoside action in the regulation of the inflammatory response remains largely unknown, especially in the DSS-induced colitis model. Various animal models of experimental IBD have been developed to examine the pathogenesis. The administration of DSS or trinitrobenzene sulfonic acid (TNBS) induces a reversible form of colitis in mice [15, 16]; this model is characterized by acute tissue inflammation in the colon and mimics the pathology of human ulcerative colitis [17].

The inflammatory response is modulated by posttranscriptional regulation of mRNA stability and translation [18]. Posttranscriptional control of inflammatory transcripts is strongly dependent on AU-rich element- (ARE-) mediated mechanisms regulated by ARE-binding proteins [19]. Tristetraprolin (TTP) is an ARE-binding protein that promotes degradation of proinflammatory mediators, such as TNF-*α*, GM-CSF, IL-2, IL-3, IL-6, CCL2, CCL3, iNOS, and COX-2 [20–26]. The upregulation of TTP mRNA can be induced by arthritis, autoimmune dysfunction, and myeloid hyperplasia, demonstrating the importance of TTP in limiting the inflammatory response [27]. During inflammation, TTP causes mRNA decay of several proinflammatory genes, such as TNF-*α*, IL-6, CCL2, and CCL3 [28]. Also, TTP has a critical effect on the anti-inflammatory response in DSS-induced colitis [29].

Here, we showed that pterostilbene 4′-*β*-glucoside is more potent than pterostilbene in mediating the anti-inflammatory effect resulting from the induction of TTP, as well as in suppressing proinflammatory cytokines, such as TNF-*α* and IL-6. Therefore, we suggest that pterostilbene 4′-*β*-glucoside has powerful therapeutic effects in IBD.

## 2. Materials and Methods

### 2.1. Reagents and Chemicals

4-PG was obtained from the Okayama University of Science Dept. of Life Science. Pterostilbene was purchased from Tokyo Chemical Industry (TCI), Japan. 4-PG and pterostilbene were dissolved in DMSO. For in vitro, 1 *μ*l (20 mM of 4-PG or pterostilbene in DMSO) was treated in 2 ml of medium on 6-well plate. For in vivo, mice were injected i.p. with 100 *μ*l of pterostilbene (2 mg/ml) to make a final 10 mg/kg. Lipopolysaccharides (LPS) and antibodies against TTP were purchased from Sigma-Aldrich (USA). Antibodies against *β*-actin were purchased from Cell Signaling. DSS was purchased from MP Biomedicals. N-acetyl-L-cysteine was purchased from Sigma-Aldrich (St. Louis, MO, USA).

### 2.2. Cells

Mouse macrophage RAW264.7 cells were cultured in DMEM supplemented with 10% FBS and 1% penicillin-streptomycin. Cell cultures were grown at 37°C in humidified incubators containing an atmosphere of 5% CO_2_. Bone marrow-derived macrophage (BMDM) was isolated from 10-week-old *Ttp^+/+^* and *Ttp^−/−^* mice. After sacrificing the mice, the femora and tibiae were carefully taken out and dissected free of adherent soft tissue. Bone marrow cells were collected by flushing the cavity by slowly injecting Hanks' balanced salt solution (HBSS) (Gibco, Grand Island, NY, USA). Then, collected cells were cultured in DMEM, and 50 ng/ml mouse macrophage colony-stimulating factor (M-CSF, PeproTech, Rocky Hill, NJ) was added to differentiate BMDMs. Three days later, nonadherent cells were removed and adherent cells were cultured in fresh DMEM for mRNA stability assay.

### 2.3. Animals and Dextran Sulfate Sodium- (DSS-) Induced Colitis

TTP knockout (KO) mice were provided by Dr. Perry J. Blackshear (Laboratory of Signal Transduction, National Institute of Environmental Health Sciences, USA). Animals were maintained in a specific pathogen-free facility. Animal studies were approved by the University of Ulsan Animal Care and Use Committee. The mice were maintained under specific pathogen-free conditions at 18–24°C and 40–70% humidity, with a 12 h light-dark cycle. Food and drinking water were available ad libitum. *Ttp*^+/+^ mice (10 weeks, *n* = 24) and *Ttp*^−/−^ male mice (10 weeks, *n* = 24) were randomly assigned to four groups (CON, DSS, DSS+ 4-PG, and 4-PG, six mice in each group for the two genotypes). To construct an acute colitis animal model, 3% DSS dissolved in drinking water was initially administered to *Ttp*^+/+^ mice and *Ttp*^−/−^ mice for 7 days in the presence or absence of 4-PG (10 mg/kg, i.p. injection). Then, the mice from the 4-PG and DSS+ 4-PG groups were given 4-PG (10 mg/kg, i.p. injection) for every day for 3 more days. After 10 days of administration, the mice were sacrificed and colon tissues, as well as serum for histology and molecular assays, were collected.

### 2.4. Measurement of Colon Length and Colon Histology

The mice were sacrificed at designated time points, the entire colon was dissected and then directly imaged in a light imaging box illuminated by fiber optic lighting (Lightools Research Inc., Encinitas, CA), and the colon length was determined using a measuring scale. To detect the pathological changes, colon tissues were fixed in 10% neutral-buffered formation solution and then dehydrated in graded alcohol, embedded in paraffin, sectioned into 4 *μ*m thick sections, and stained with hematoxylin and eosin.

### 2.5. Reverse Transcription-Polymerase Chain Reaction (RT-PCR) and Real-Time Quantitative PCR

Total RNA was extracted from RAW264.7 cells and colon tissues using TRIzol reagent (Invitrogen), according to the manufacturer's instructions. In brief, 2 *μ*g of total RNA was used to generate cDNA using M-MLV reverse transcriptase (Promega Corporation, WI, USA). The resulting cDNA was subjected to PCR to amplify mouse GAPDH (f-aggccggtgctgagtatgtc, r-tgcctgcttcaccttct, 530 bp), mouse 18S RNA (forward, 5′-cagtgaaactgcgaatggct-3′, reverse, 5′-tgccttccttggatgtggta-3′, 397 bp), mouse TNF-*α* (f-agcccacgtcgtagcaaaccaccaa, r-acacccattcccttcacagagcaat, 421 bp), mouse TTP (f-ctctgccatctacgagagcc, r-gatggagtccgagtttatgttcc, 105 bp), and mouse IL-6 (f-gtggaaatgagaaaagagttgt, r-cctcttggttgaagatatgaat, 283 bp). Real-time quantitative PCR was performed with SYBR Green qPCR Master Mix (2x, USB Production; Affymetrix) on an ABI 7500 Fast Real-Time PCR System (Applied Biosystems, Carlsbad, CA). the real-time PCR primer pairs were as follows: mouse TTP (forward, 5′-ccaggctggctttgaactca-3′, reverse, 5′-acctgtaaccccagaacttgga-3′), and mouse GAPDH (forward, 5′-gggaagcccatcaccatct-3′, reverse, 5′-cggcctcaccccatttg-3′).

### 2.6. Western Blot Assays

Cell lysates and colon tissues were prepared using RIPA buffer containing protease inhibitors and phosphatase inhibitors, and the total protein concentration of the lysates was measured using a BCA protein assay kit (Pierce Biotechnology, Rockford, IL, USA). Proteins were resolved by SDS-PAGE, transferred onto polyvinylidene difluoride membrane, and probed with appropriate dilutions of the following antibodies: anti-*β*-actin (Cell Signaling, 4967S, 1 : 2000 dilution) and anti-TTP (Sigma, T5327, 1 : 2000 dilution). Immunoreactivity was detected using the ECL detection system (GE Healthcare Bio-Sciences, Little Chalfont, UK). The relative band density was analyzed using ImageJ software (U.S. National Institutes of Health, Bethesda, MD).

### 2.7. Transfection

For knockdown of TTP gene expression, RAW264.7 cells were transfected with predesigned siRNA against mouse TTP (sc-36761, Santa Cruz Biotechnology) using the Lipofectamine 2000 method, according to the manufacturer's protocol (Invitrogen). Cells were treated with 10 *μ*M 4-PG in the presence or absence of 100 ng/ml LPS for 10 h. The decrease in TTP expression was confirmed by reverse transcription-polymerase chain reaction. The expression levels of TNF-*α* mRNA and protein were analyzed by reverse transcription-polymerase chain and ELISA, respectively.

### 2.8. ELISA

Supernatants were collected from cultured RAW264.7 cells, which were pretreated with 4-PG (10 *μ*M) for 6 h followed by the stimulation with LPS (100 ng/ml) for 4 h. Serum was collected from mice (*n* = 6) sacrificed in each group and kept on ice. Then, the levels of TNF-*α* and IL-6 proteins in the supernatants and serum samples were analyzed using BioLegend MAX™ ELISA kits (BioLegend, San Diego, USA).

### 2.9. Myeloperoxidase (MPO) Assay

MPO enzyme activity in colon tissues was measured using the Mouse Myeloperoxidase DuoSet kit (R&D Systems, Minneapolis, MN).

### 2.10. ROS Measurement by Flow Cytometry

RAW264.7 cells were preincubated with or without 4-PG (10 *μ*M) for 6 h and N-acetyl-L-cysteine (NAC, 1 mM) for 30 min followed by the stimulation of LPS (100 ng/ml) for another 4 h. Then, medium was removed and cells were incubated in 1x PBS containing 5 *μ*M of CM-H_2_DCFDA (C6827, Invitrogen) for 30 min at 37°C. The measurement of intracellular ROS was performed using a flow cytometer with fluorescence-activated cell sorter (FACSCanto II) and analyzed by FlowJo V10 software (Tree Star Inc., San Carlos, CA).

### 2.11. Statistical Analysis

All values are expressed as mean ± SEM. Student's *t*-test was used to evaluate the differences between the samples of interest and the corresponding controls. The differences between groups were assessed by one-way ANOVA.

## 3. Results

### 3.1. 4-PG Induces TTP mRNA and Protein Expression in RAW264.7 Cells

**Figure 1:**
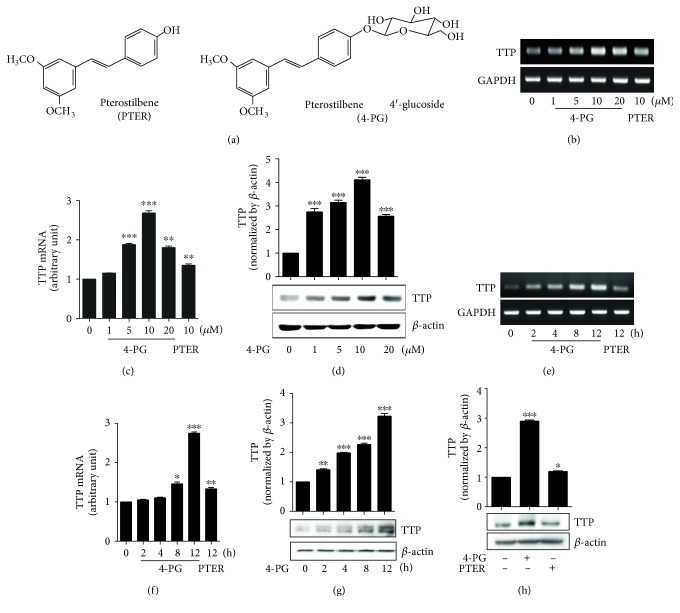
4-PG induces TTP mRNA and protein in RAW264.7 cells. (a) Chemical structure of pterostilbene (PTER) and pterostilbene 4′-glucoside (4-PG). (b–d) RAW264.7 cells were treated at various concentrations of 4-PG (0, 1, 5, 10, and 20 *μ*M) and PTER (10 *μ*M) for 12 h. The mRNA and protein expression of TTP were determined by RT-PCR (b), real-time PCR (c) and Western blot (d), respectively. (e–g) RAW264.7 cells were treated with 4-PG (10 *μ*M) and PTER (10 *μ*M) in indicated time points (0, 2, 4, 8, and 12 h). The mRNA and protein expression of TTP were analyzed by RT-PCR (e), real-time PCR (f) as well as Western blot (g), respectively. (h) To compare the effect of 4-PG and PTER on TTP protein expression, RAW264.7 cells were treated with 4-PG (10 *μ*M) and PTER (10 *μ*M) for 12 h. The protein level of TTP was measured by Western blot assay. ImageJ software was used for densitometry analysis. GAPDH and *β*-actin were used as internal controls. Data are expressed as means ± SE, *n* = 3. ^∗^: *P* < 0.05, ^∗∗^: *P* < 0.01, ^∗∗∗^: *P* < 0.001 versus cells without treatment.

Accumulating evidence indicates that the 4-PG exhibits a chemopreventive effect in cancer, allergies, and Alzheimer's disease [12]. However, the exact mechanisms of 4-PG action in various diseases are still largely unknown. First, to investigate the mechanisms of 4-PG associated with the inflammatory response, the expression of TTP in RAW264.7 murine macrophage cells was measured after treatment with 4-PG at various concentrations (0, 1, 5, 10, and 20 *μ*M) and at different time points (0, 2, 4, 8, and 12 h). 4-PG increased TTP mRNA and protein levels in dose- and time-dependent manners. The optimal level of TTP was observed in RAW264.7 cells treated with 10 *μ*M 4-PG for 12 h (Figures 1(a), 1(b), 1(c), 1(d), 1(e), and 1(f)). Also, in cell viability assay, there was no effect on cell growth in the concentration of 4-PG (10 *μ*M) (data not shown). Therefore, the concentration of 4-PG was used with 10 *μ*M for in vitro experiments. Moreover, a number of recent reports have indicated that pterostilbene showed anti-inflammatory, antioxidant, and anticarcinogenic properties [7, 30]. To evaluate whether 4-PG induces higher TTP expression than pterostilbene, we treated RAW264.7 cells with 4-PG and pterostilbene for the same length of time (12 h) and with the concentration (10 *μ*M). As shown in Figure 1, 4-PG induced significantly more TTP than pterostilbene, both at the transcriptional and translational levels. Taken together, these data demonstrated that 4-PG has the potential to increase the expression level of TTP and that the anti-inflammatory property of 4-PG may be mediated by enhanced TTP.

### 3.2. 4-PG Decreases LPS-Induced Proinflammatory Cytokines via Increased TTP in RAW264.7 Cells

**Figure 2:**
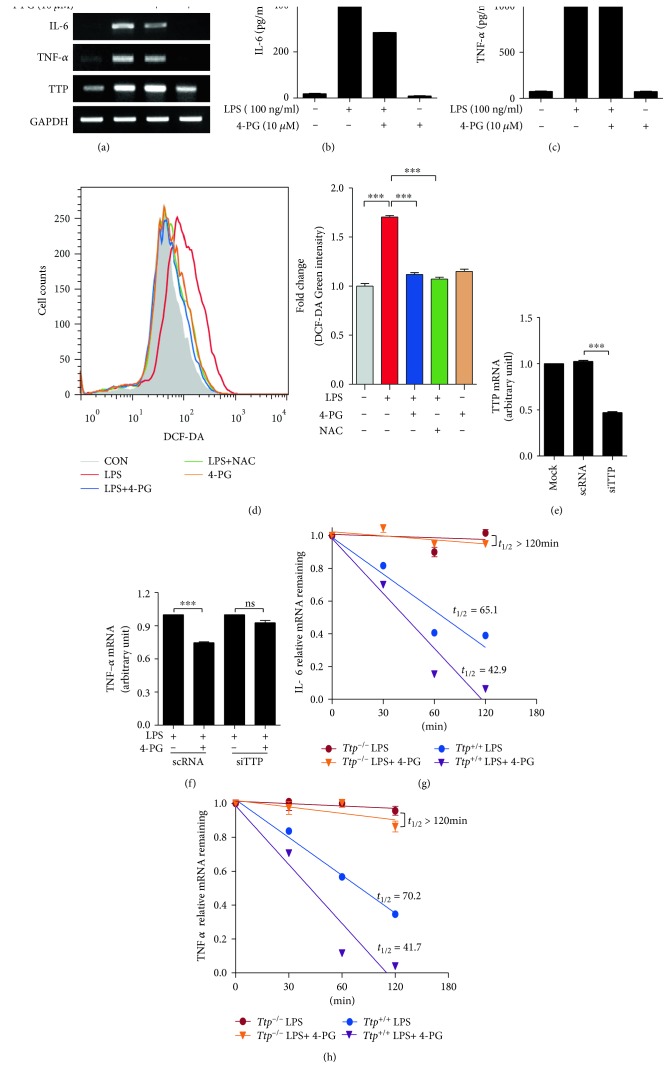
4-PG decreases LPS-induced proinflammatory cytokine expression via enhancement of TTP in RAW264.7 cells. RAW264.7 cells were pretreated with 4-PG (10 *μ*M) for 6 h followed by the stimulation of LPS (100 ng/ml) for 4 h. (a) The mRNA expression of IL-6, TNF-*α*, and TTP was measured by RT-PCR. (b, c) The secreted protein level of IL-6 and TNF-*α* in the supernatant of cultured cells was determined by ELISA. (d) Effects of 4-PG on reactive oxygen species (ROS) production were detected in RAW 264.7 cells. RAW264.7 cells were preincubated with or without 4-PG (10 *μ*M) for 6 h and N-acetyl-L-cysteine (NAC, 1 mM) for 30 min followed by the stimulation of LPS (100 ng/ml) for another 4 h. The intracellular level of ROS was stained by H_2_DCFDA, and the production of ROS was measured by flow cytometry. The fold change of fluorescence intensity is presented as means ± SE, *n* = 3. ^∗∗∗^: *P* < 0.001. To confirm that the anti-inflammatory effect of 4-PG was mediated by TTP, RAW264.7 cells were transfected with scramble RNA (scRNA) and siRNA against TTP (siTTP) for 24 hours. Then, cells were pretreated with 4-PG (10 *μ*M) for 6 h followed by the challenge of LPS (100 ng/ml) for 4 h. (e, f) The mRNA expression of TTP and TNF-*α* was analyzed by real-time PCR, respectively. GAPDH was used as internal control. (g, h) To determine the mRNA-decaying activity of TTP on inflammatory cytokines, bone marrow-derived macrophage (BMDM) extracted from *Ttp^+/+^* and *Ttp^−/−^* mice was pretreated with 4-PG (10 *μ*M) for 6 h followed by the stimulation of LPS (100 ng/ml) for 4 h. Then, the mRNA level of IL-6 (g) and TNF-*α* (h) was measured by real-time PCR at indicated times (0, 30, 60, and 120 min) after the addition of 5 *μ*g/ml actinomycin D. Data are expressed as means ± SE, *n* = 3. ^∗∗^: *P* < 0.01, ^∗∗∗^: *P* < 0.001, and ns: not significant.

Posttranscriptional control of inflammatory transcripts is strongly dependent on ARE-binding proteins, especially TTP, which promote the degradation of proinflammatory cytokines. To determine whether the anti-inflammatory effect of 4-PG is mediated by TTP, RAW264.7 cells were incubated with 4-PG (10 *μ*M) for 6 hour, followed by stimulation with LPS (100 ng/ml) for another 4 h. Our results revealed that the increases in the mRNA and protein levels of proinflammatory cytokine (TNF-*α* and IL-6) induced by LPS were significantly downregulated by 4-PG (Figure 2(a), 2(b), and (c)). The treatment of 4-PG alone had no effect on the production of TNF-*α* and IL-6 in RAW264.7 cells. In addition, to assess the antioxidant effect of 4-PG, ROS levels were detected with H_2_DCFDA. The LPS-induced ROS levels were suppressed by 4-PG treatment (Figure 2(d)). N-Acetyl-L-cysteine, antioxidant as positive control, also reduced the LPS-induced ROS levels. To evaluate whether the downregulation of TNF-*α* and IL-6 by 4-PG was associated with increased TTP, we transfected RAW264.7 cells with either scramble RNA or siRNA against TTP for 24 hours. As shown in Figure 2(e), TTP mRNA decreased by more than 50% in cells transfected with TTP siRNA relative to cells transfected with control siRNA. Additionally, the treatment of siRNA-transfected cells with 4-PG did not show any significant decrease in the level of TNF-*α* mRNA (Figure 2(f)). Next, we analyzed the ability of 4-PG to enhance degradation of LPS-induced inflammatory cytokine mRNAs in BMDM cells of *Ttp*^+/+^ mice by calculating the half-lives of these mRNAs. Although the half-lives of IL-6 and TNF-*α* were 65.1 minutes and 70.2 minutes, respectively, in BMDM cells from *Ttp^+/+^* mice with LPS alone (Figures 2(g) and 2(h)), these half-lives in BMDM cells from *Ttp^+/+^* mice with both 4-PG and LPS were reduced to 42.9 and 41.7 minutes, respectively (Figures 2(g) and 2(h)). However, in BMDM cells from *Ttp*^−/−^mice, the half-lives of IL-6 and TNF-*α* were >120 min after actinomycin D and 4-PG did not enhance the decay of LPS-induced IL-6 and TNF-*α* mRNAs (Figures 2(g) and 2(h)). These results indicate that *Ttp*-mediated destabilization of inflammatory cytokine mRNAs contributes to anti-inflammatory function of 4-PG. Taken together, these data demonstrated that TTP plays an important role in the anti-inflammatory effect of 4-PG.

### 3.3. TTP Deficiency Abolishes the Anti-Inflammatory Effect of 4-PG in a DSS-Induced Colitis Model

**Figure 3:**
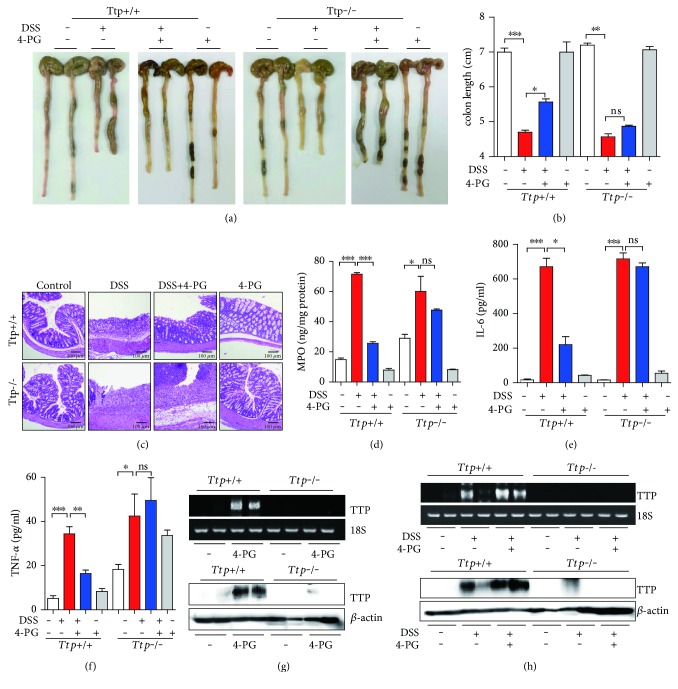
TTP deficiency abolished the anti-inflammatory effect of 4-PG in a DSS-induced colitis model. To construct an acute colitis animal model, 10 weeks *Ttp^+/+^* and *Ttp^−/−^* mice were administrated with 3% DSS dissolved in drinking water for 7 days in the presence or absence of 4-PG (10 mg/kg, i.p. injection). Then, the mice from 4-PG and DSS+ 4-PG groups were still administrated with 4-PG (10 mg/kg, i.p. injection) for another 3 days. After 10 days' treatment, the mice were sacrificed, and the entire colon was removed from the cecum to the anus. (a) Representative images of 6 tests conducted in each group. (b) Bar graph represents the colon length from each group. (c) Colon tissues were excised, and representative colon histology was shown by hematoxylin and eosin staining. (d) Neutrophil infiltration into the colon was measured by the expression of myeloperoxide (MPO) in colon tissues. The protein level of proinflammatory cytokines, IL-6 (e) and TNF-*α* (f), were detected by ELISA kit from serum. (g, h) The mRNA and protein levels of TTP in colon tissues from *Ttp^+/+^* and *Ttp^−/−^* mice were detected by RT-PCR and Western blot. 18S RNA and *β*-actin were used as internal controls. Data are expressed as means ± SE, *n* = 6. ^∗^: *P* < 0.05, ^∗∗^: *P* < 0.01, ^∗∗∗^: *P* < 0.001, and ns: not significant.

In a previous study, we proved that TTP plays an important role in anti-inflammatory response in DSS-induced colitis [29]. To confirm that the anti-inflammatory effect of 4-PG was mediated by TTP in vivo, we constructed an acute colitis animal model. 10-week-old wild-type and TTP systemic knockout mice were treated with 3% DSS (*w/v*) in the presence or absence of 4-PG (10 mg/kg, i.p. injection) for 7 days; additionally, the mice from the 4-PG and DSS+ 4-PG groups were further treated with 4-PG (10 mg/kg, i.p. injection) over the following 3 days. After 10 days, the mice were sacrificed and the entire colon was removed from the cecum to the anus. According to our results, DSS induced a shortening of the colon, which was improved by the combinatorial treatment with 4-PG in *Ttp^+/+^* mice (colon length, DSS: 4.70 ± 0.10 cm versus DSS+ 4-PG: 5.55 ± 0.15 cm, Figures 3(a) and 3(b)). However, in TTP-deficient mice, no significant difference was observed in colon lengths between the DSS and DSS+ 4-PG groups (colon length, DSS: 4.55 ± 0.15 cm versus DSS+ 4-PG: 4.85 ± 0.05 cm, Figures 3(a) and 3(b)). Furthermore, to evaluate the histological changes in colon tissues, H&E staining was performed. Consistent with previous reports, DSS treatment resulted in a great loss of epithelial crypts and massive infiltration of inflammatory cells into the mucosa in both *Ttp^+/+^* mice and *Ttp*^−/−^mice. The severity of colon tissue pathology was partly mitigated by 4-PG treatment in *Ttp^+/+^* mice, while in TTP-deficient mouse, the protective effect of 4-PG was abolished (Figure 3(c)). Due to the variety of types of inflammatory cells that had infiltrated into the mucosa under the challenge of DSS, we next evaluated MPO activity as a marker for neutrophil infiltration in colon tissues. Although 4-PG reduced the MPO activity induced by DSS in *Ttp^+/+^* mice, in *Ttp*^−/−^mice, 4-PG showed no beneficial effect on inhibition of MPO activity (Figure 3(d)). On the other hand, we also evaluated the levels of IL-6 and TNF-*α* protein in serum by ELISA. 4-PG significantly decreased the expression of IL-6 and TNF-*α* increased by DSS in *Ttp^+/+^* mice. However, TTP-deficient mice showed no decrease in proinflammatory cytokines upon treatment with 4-PG (Figures 3(e) and 3(f)). The only 4-PG-treated mice had no effect on the production of IL-6 and TNF-*α* in *Ttp^+/+^* mice and *Ttp*^−/−^mice (Figures 3(e) and 3(f)). To confirm the levels of TTP expression in colon tissues from *Ttp^+/+^* mice and *Ttp*^−/−^mice, we performed the RT-PCR and Western blot analysis (Figures 3(g) and 3(h)). The administration of 4-PG induced the expression of TTP mRNA and protein in colon tissues from *Ttp^+/+^* mice but not in colon tissues from *Ttp*^−/−^mice. Taken together, these data clearly demonstrated that the beneficial effect of 4-PG on DSS-induced inflammatory colitis was strongly related to TTP expression and that TTP also plays a pivotal role in the anti-inflammatory effect of 4-PG.

## 4. Discussion

Pterostilbene, a natural derivative of resveratrol, has been shown to possess antioxidant, antiproliferative, anticancer, and pain-relieving activities both in vitro and in vivo [31]. However, the effect of the glycosylation of pterostilbene on inflammatory responses has not been well studied. In the present study, we provide evidence that pterostilbene 4′-*β*-glucoside (4-PG) exerts its anti-inflammatory effects on DSS-induced colitis by modulating TTP expression in colon tissues and in macrophage cell line. Our results demonstrated that 4-PG increases TTP expression at both the transcriptional and translational levels (Figure 1), thereby decreasing the expression of proinflammatory cytokines induced by LPS (Figure 2). However, in TTP-deficient mice or in macrophages transfected with TTP siRNA, 4-PG fails to block the LPS- and DSS-induced inflammatory responses (Figures 2 and 3). In addition, 4-PG has the antioxidative effect by suppressing the ROS levels (Figure 2). 4-PG-like pterstilbene may decrease the level of ROS via increasing antioxidant enzymes such as superoxide dismutase-1(SOD1) and peroxiredoxin-4 (PRDX4) [32]. The bioavailable concentration of pterostilbene is critical in the protection of melanoma and pancreatic cancer [33]. According to Benlloch et al. [33], a half-life of pterostilbene in circulating plasma after administration of i.v. is 70–73 min. Based on their results, the beneficial effects of 4-PG in DSS-induced colitis may be in the charge of 4-PG metabolites by indirect mechanisms. Also, the increase of bioavailability of curcumin when cotreated with emu oil and curcumin improves anti-inflammatory potential of curcumin [34]. Thus, the bioavailability of 4-PG should be considered to achieve higher effects in DSS-induced colitis. Even though the concentration of 4-PG in plasma was not measured in 4-PG-treated (10 mg/kg) mice, the increase of TTP levels and anti-inflammatory response in 4-PG-treated (10 mg/kg) mice was investigated in Figures 3(e) and 3(f). Therefore, we suggest that 4-PG initiated the anti-inflammatory response via the activation of the TTP-mediated signal pathway in DSS-induced colitis. It is also possible that other indirect mechanisms may be responsible of TTP-mediated anti-inflammatory response in vivo [33]. However, we did not analyze the mechanisms of 4-PG-induced TTP expression; elucidation of this will require additional studies.

TNF-*α* and IL-6 are both central proinflammatory cytokines associated with the development of intestinal inflammation in animal colitis models [35] and human IBD [36]. TNF-*α* plays a central role in initiating and regulating the cytokine cascade during inflammatory responses. As a multifunctional cytokine, IL-6 exhibits major regulatory effects on the inflammatory process [37]. The mRNAs of these proinflammatory cytokines contain AREs within their 3′-untranslated region (3′-UTR), and their expression can be downregulated by TTP. Thus, TTP-deficient mice experienced more severe tissue damage in the colon compared to wild-type mice during the challenge of DSS. Additionally, in TTP KO mice, the reduction effect of 4-PG in downregulating the production of inflammatory cytokines was lost (Figure 3). On the other hand, it has been reported that LPS can rapidly and strongly increase the expression of TTP [38, 39]. Consistent with a previous study, we observed that treatment with LPS alone increased TTP mRNA expression in mouse RAW264.7 cells with 4 hours posttreatment, although its effect was mild. Additionally, we found that treatment with 4-PG before stimulation with LPS increased TTP expression more than individual treatment with LPS and significantly decreased the production of TNF-*α* and IL-6 (Figure 2). Accumulating evidence suggests that although LPS increases the expression level of TTP, it also activates p38 MAPK, which results in inactivation of TTP and allows for stabilization of ARE-containing TNF-*α* and IL-6 mRNAs [40, 41]. Thus, further study is needed to test that the anti-inflammatory property of 4-PG does not only result in increased expression of TTP but also result in inhibition of p38, which subsequently enhances the activity of TTP in promoting the degradation of proinflammatory cytokines. Our data clearly indicate that TTP is a pivotal regulator of intestinal inflammatory disease and that 4-PG mediates an anti-inflammatory function via increases in TTP expression.

In conclusion, the data presented here indicate that the anti-inflammatory activity of 4-PG was mediated by induction of TTP, which resulted in downregulation of inflammatory cytokines such as TNF-*α* and IL-6. TTP deficiency worsened DSS-induced colitis and abrogated the reduction effect of 4-PG against colitis. Taken together, these data demonstrated for the first time that 4-PG-induced TTP plays a critical role in reducing the DSS-induced colitis, which may be an attractive candidate for treatment of IBD.
